# Book Reviews

**Published:** 1872-11-15

**Authors:** 


### Book Reviews.

Ovarian Tumors. Their Pathology, Diagnosis and Treatment, especially by Ovariotomy. By E. Randolph Peaslee, M. D., LL. D., etc.; with fifty-six illustrations on wood. D. Appleton & Co., New York.

This is a volume of 538 pages, the first 222 pages being devoted to the normal and pathological anatomy of the ovaries, and the pathology, diagnosis, and treatment of ovarian tumors, excepting by ovariotomy. The remainder of the work is taken up exclusively with a full consideration of the subject of ovariotomy, including its history, statistics, practical details, and after treatment. The author aims to decide all practical questions by the aggregate experience of all ovariotomists up to the present time.

The Transactions of the American Medical Association. Vol. 23, 1872. Collins, 705 Jayne street, Philadelphia.

This comprises a volume of 750 pages, made up of the usual assortment of valuable and interesting reports and essays. A Nomenclature of Diseases, with the reports of the majority and of the minority of the committee thereon, forms an appendix of 94 pages at the end of the volume.

We acknowledge the receipt of Lindsay & Blakiston's "Physician's Visiting List" for 1873, the twenty-second year of its publication. We have always found it to be the most useful and convenient book of the kind published.

The Anatomy and Development of Rodent Ulcer: A Boylston Medical Prize Essay for 1872. By J. Collins Warren, M. D. Boston: Little, Brown & Co.

A monograph of 65 pages, with colored plates.

Treatment of Strictures of the Urethra by Laminaria Digitata and Galvanism. By Robert Newman, M. D. New York.

A paper read before the Medical Journal Association of New York.

A Clinical Analysis of the Inflammatory Affections of the Inner Ear. By H. Knapp, Surgeon to the New York Ophthalmic and Aural Institute. New York: Williams, Wood & Co., publishers. Pamphlet—73 pages.

